# Outcomes of First-Line Chemotherapy in Patients with Advanced or Metastatic Leiomyosarcoma of Uterine and Non-Uterine Origin

**DOI:** 10.1155/2009/348910

**Published:** 2009-12-29

**Authors:** A. W. Oosten, C. Seynaeve, P. I. M. Schmitz, M. A. den Bakker, J. Verweij, S. Sleijfer

**Affiliations:** ^1^Department of Medical Oncology, Erasmus University Medical Center, Daniel den Hoed Cancer Center, Groene Hilledijk 301, 3075 EA Rotterdam, The Netherlands; ^2^Department of Biostatistics, Erasmus University Medical Center, Daniel den Hoed Cancer Center, Groene Hilledijk 301, 3075 EA Rotterdam, The Netherlands; ^3^Department of Pathology, Erasmus University Medical Center, Josephine Nefkens Institute, 3000 CA Rotterdam, The Netherlands

## Abstract

Although leiomyosarcomas (LMSs) form the largest subgroup of soft tissue sarcomas (STSs), the efficacy of chemotherapy in this group is largely unclear, partly because older studies are contaminated with gastrointestinal stromal tumors (GISTs). In this retrospective study we investigated the outcome of first line chemotherapy in 65 patients with unresectable or metastatic LMS. The overall response rate (ORR) was 18%; and the median progression-free (PFS) and overall survival (OS) were 3.8 and 9.7 months respectively. No statistically significant differences in outcomes for uterine and non-uterine LMS were found. In non-uterine LMS, however, the PFS and OS seemed to be longer for females than for males, potentially negatively affecting outcomes in this group. If our observations are confirmed in other series, they would suggest that studies performed in STS patients should not only stratify for histological subtype but also for uterine versus non-uterine LMS and for gender.

## 1. Introduction

Adult soft tissue sarcomas (STSs) are a heterogeneous and rare group of tumors accounting for only 1% of all cancers. This group comprises over 50 different tumor entities with considerable differences in terms of clinical behaviour and genetic variances. Despite improvements in treatment over the past years, many patients present with locally advanced or metastatic disease and are therefore not amenable to surgical therapy. For these patients palliative chemotherapy is an option. With the exception of gastrointestinal stromal tumors (GISTs) and small blue round cell sarcomas such as Ewing-like tumors and rhabdomyosarcomas, systemic chemotherapy for the different STS subtypes is largely similar, with doxorubicin (DOX) and ifosfamide (IFS) being the backbone of treatment [1, 2]. In recent years, however, the insight has emerged that systemic therapy should become more tailored, for STS in particular according to the histological subtype. The correctness of this approach is clearly illustrated by the remarkable efficacy of imatinib in patients with advanced GISTs and dermatofibrosarcoma protuberans, while other subtypes of STS do not respond to imatinib at all [3–5]. Other examples include the activity of taxanes in patients with metastatic angiosarcoma, in particular those originating in the head and neck region [6–8] and of trabectidin in patients with myxoid/round cell liposarcomas [9].

A large subgroup of STSs accounting for approximately 24% of all STSs is the group of leiomyosarcomas (LMSs) [10]. LMS is a mesenchymal tumor of smooth muscle origin found in the uterus, gastrointestinal tract, or deep soft tissue. Cutaneous LMS is a site-specific subtype of LMS with a very good prognosis. Outcomes obtained by systemic therapy for LMS are rather poor compared to the efficacy in other STS subtypes [11, 12]. However, since GIST resembles LMS histopathologically and only recently can be reliably distinguished from LMS, many GISTs have been diagnosed as LMSs in older series. As it is known at the moment that GISTs are almost completely resistant to standard cytotoxic chemotherapy [13], the efficacy of chemotherapy in pure LMS, therefore, requires further investigation. Additionally, within the group of noncutaneous LMS, two main categories can be distinguished: tumors arising from the uterus versus non-uterine LMS. A recent study reported differences in gene expression patterns between uterine and non-uterine LMS [14]. Moreover, in contrast to other STS entities, uterine LMSs seem to be in particular sensitive to the combination of gemcitabine and docetaxel [15].

Consequently, more insight into the outcome of chemotherapy in LMS as well as further research into possible differences between uterine and non-uterine LMS is needed. This retrospective analysis aimed to describe the activity and outcome of chemotherapy in patients with advanced/metastatic LMS and to assess whether there are differences between LMS originating in the uterus versus LMS originating elsewhere.

## 2. Patients and Methods

From the institutional database of the Erasmus MC-Daniel den Hoed, a tertiary cancer centre in Rotterdam, The Netherlands, we selected patients with leiomyosarcoma who were treated with first-line chemotherapy for locally advanced or metastatic disease. Patients who received only adjuvant chemotherapy were not included in the analysis. Followup data were available until the 1st of December 2007.

Clinical and demographical data were collected by reviewing patient files. Recorded baseline characteristics included sex, age at diagnosis, site of primary LMS, histological subtype (if available), sites of metastases, and WHO performance status at the start of chemotherapy. Furthermore, data were recorded on type of response and PFS (see below). Survival data were collected from the charts and, if missing, by contacting the general practitioner or by checking the municipal registries.

For all patients, histological examination was performed at our hospital by specialised soft tissue pathologists either after primary surgery or upon revision after referral. For the purpose of this analysis, revision of histological material was repeated in those patients in whom GIST was suspected on clinical grounds. Soft tissue leiomyosarcomas were graded using the FNCLCC (Fédération Nationale de Centres de Lutte Contre le Cancer) system. Grade 1 tumors were defined as low grade, grades 2 and 3 tumors as high grade. Uterine leiomyosarcomas were defined as either high or low grade according to mitotic activity and necrosis.

All radiological examinations were performed at our hospital. Patients underwent a baseline CT scan before starting chemotherapy, and evaluation CT-scanning was performed after each 2 or 3 cycles of chemotherapy in most patients. Response was evaluated according to the WHO criteria in those patients treated before 2000 and according to the Response Evaluation Criteria In Solid Tumours (RECIST) criteria [16] for those patients treated beyond 2000. Outcomes are known to be similar in STSs with either criteria [17].

PFS was defined as the time (months) between start of chemotherapy until disease progression or death, whatever came first. OS was defined as the time (months) between start of chemotherapy until death from any cause.

Patients who were treated with the goal of making locally advanced disease amendable for surgical resection (induction chemotherapy) were excluded from analysis of progression free and overall survival but were included for analysis of response rates.

## 3. Statistical Analyses

Response rates, median progression free survival (PFS), and overall survival (OS) from the start of chemotherapy are reported for the whole group of STS and were compared between uterine LMS and LMS originating elsewhere. Percentages were compared with chi-square tests. Analysis of PFS and OS was performed with Kaplan-Meier curves and the log rank test.

## 4. Results

Sixty-nine patients with LMS who received chemotherapy between January 1991 and August 2007 were identified. After exclusion of two patients who were treated with adjuvant chemotherapy and two patients who were found to have a GIST on revision, 65 patients with locally advanced or metastatic disease remained eligible and were included in the analysis.

At the time of analysis, 56 patients had died; 7 patients remained alive. For 2 patients it was reasonable to believe that they had died, but this could not be confirmed (no data were available in the municipal registry, and it was assumed that both patients died abroad).

Patient characteristics of the two groups, non-uterine (*n* = 32) and uterine LMS (*n* = 33), respectively, are given in Table 1. Except for the obvious gender difference, there were no significant differences between both groups.

Fifty-five patients had distant metastases, of whom 16 patients also had a relapse at the primary site, and 10 patients had locally advanced disease without signs of distant metastases. Of the latter patients, 9 received chemotherapy with the aim to decrease tumor size potentially allowing a subsequent resection (induction treatment), while for one patient the intention of treatment was palliation because the clinical condition was considered too poor (WHO PS 2) to undergo subsequent surgery.

Treatment characteristics are described in Table 2. Because in our hospital, STS patients are preferentially treated in the context of studies conducted by the European Organisation of Research and Treatment of Cancer-Soft Tissue and Bone Sarcoma Group (EORTC-STBSG), a wide variety of chemotherapeutic regimens were used over the years. There were no significant differences in administered chemotherapeutic regimens between the patients with uterine versus non-uterine LMS.

**Figure 1:**
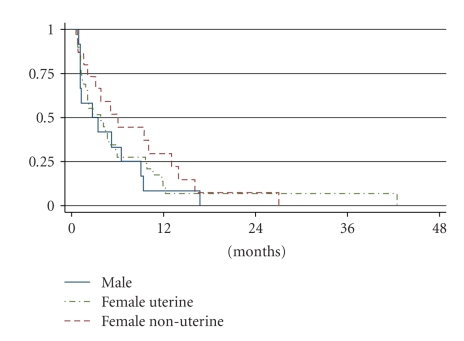
Progression free survival according to gender and site of origin.

**Figure 2:**
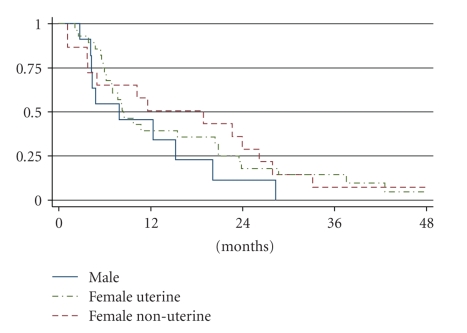
Overall survival according to gender and site of origin.

Overall, the RR (RR, = complete (CR) + partial response (PR)) was 18% in the patients evaluable for response (*n* = 64, see Table 3), while for the 56 patients evaluable for survival, the median PFS and OS were 3.8 months and 9.7 months, respectively. Dissecting non-uterine from uterine LMS, the overall response rates (CR + PR) were 22% and 15%, respectively (Table 3) (*P* = .26). Median PFS and OS in non-uterine LMS was 5.1 and 11.6 months, respectively, compared to 3.7 months and 8.3 months, respectively, in uterine LMS, which was not statistically significantly different (*P* = .76 for PFS and *P* = .74 for OS) (Figures 1 and 2). After correction for prognostic factors for the whole group (age at the start of chemotherapy, tumor size, histological subtype, and absence or presence of distant metastases and their localisation), no statistically significant differences were found.

Regarding the 27 patients with a non-uterine LMS, the PFS and OS in men (*n* = 12) were 2.7 months and 7.9 months, respectively, while these measures in women were longer being 6.0 months and 18.9 months, respectively. These differences, however, again were not statistically significant (*P* = .48 for PFS, *P* = .47 for OS). 

## 5. Discussion

In this retrospective analysis on the outcome of first-line chemotherapy in patients with advanced or metastatic LMS, we found an overall response rate (ORR) of 18% (CR + PR). Median PFS and OS were 3.8 and 9.7 months, respectively. The ORR was not significantly different in non-uterine versus uterine LMS, while there was a trend for longer PFS and OS in female patients with a non-uterine LMS as compared to men.

Although LMSs form one of the largest subgroups of soft tissue sarcomas [10], little is known about the efficacy of chemotherapy for this entity. Although previous STS studies have specified outcomes for LMS, it is likely that these studies were contaminated with GIST patients. It is only since the recent advent of immunohistochemistry, and if necessary molecular characterisation, that GIST can be reliably distinguished from LMS.

Regarding the outcome measures ORR, PFS, and OS, previous studies have shown comparable although slightly better outcomes for LMS than in our current analysis. The largest study, a retrospective analysis of 2185 patients (1922 assessable for response) with advanced STS treated in studies of the EORTC-STBSG, showed an overall response rate of 26% for the total group of STSs, compared to 22% for 492 patients with LMS. Median OS was 51 weeks for the whole group and 52 weeks for patients with LMS [12]. Another study of this group, largely containing similar cases as the former study, revealed that in terms of progression-free rates at 3 and 6 months after initiation of first-line therapy, LMS patients in general did worse than patients with other STS entities [18]. A third study, a retrospective analysis of 488 STS patients mostly treated with doxorubicine and/or ifosfamide, showed a median OS of 11 months for the LMS patients, while OS for patients with other common STS entities including synovial sarcomas, liposarcomas, and fibrosarcomas was better being 19, 18, and 14 months, respectively [19]. In all of these studies it is likely that cases of GIST were included in the LMS groups. With this caveat in mind, these studies suggest that outcomes in LMS patients are at the lowest boundary of antitumor activity achieved by first-line chemotherapy in STS.

With respect to the subgroup of LMSs, there are several indications that LMS arising in the uterus is biologically different than LMS originating elsewhere. Firstly, gene expression profiling studies suggest a small difference between uterine and other LMSs. Although the majority of expressed gene fragments are similar, about 25 discriminator genes differentiate between the two groups. These genes are primarily related to tissue type [14, 20]. Secondly, several studies suggest that uterine LMS differs with respect to sensitivity to chemotherapy compared to other STS types. While gemcitabine is considered inactive in unselected STS [21, 22], several phase II studies have shown that gemcitabine monotherapy can produce antitumor activity in uterine LMS [23–27]. Furthermore, in two phase II trials the combination of gemcitabine and docetaxel, the latter also regarded inactive in unselected STS [28], was recently found to induce remarkably high response rates of 35% to 53% in patients with advanced uterine LMS [15, 29]. Median PFS and OS were 4.4 months and 16 months, respectively, in the most recent Study of the Gynecologic Oncology Group. Also, Bay et al. retrospectively evaluated the combination of gemcitabine and docetaxel in 133 patients with STS, including 76 LMSs [30]. They found an overall response rate of 18.4%, being 24.2% for LMS and 10.4% for other STSs, respectively (*P* = .06). The median overall survival of the total group was 12.1 months, but overall survival was better for LMS patients versus patients with other histologies (*P* = .01). Response rates and survival were the same for uterine LMS versus other primary sites of LMS. Overall, although nonrandomised and retrospective studies are highly prone for selection bias, which may affect outcomes, most of the performed studies point at a difference between uterine and non-uterine LMSs. As a result, uterine LMS should be distinguished from non-uterine LMS for future studies. In our study population, in which no gemcitabine/docetaxel-containing chemotherapy was given, there were no statistically significant differences between non-uterine and uterine LMSs in terms of response rates according to WHO and RECIST, OS, and PFS. It would be worthwhile to further investigate the value of gemcitabine/docetaxel in uterine LMSs in greater groups.

Remarkably, in our analysis survival times seemed longer for females with non-uterine LMS, compared to male LMS patients (OS 18.9 versus 7.9 months, PFS 6.0 versus 2.7 months). It should be noted that these differences were not statistically significant which, however, may be due to the small numbers. This seemingly worse outcome for males with LMS, which adversely affects the outcomes of the whole group with non-uterine LMS, is in line with studies in tumor types other than STS also reporting worse outcomes for males. It is postulated that this may be due to differences in body mass index and composition and differences in drug pharmacodynamics and drug metabolism [31].

In conclusion, this study provides data on the outcome of chemotherapy for LMS patients. Although no significant differences were found for primary uterine versus non-uterine disease localisation, worse outcomes in male LMS patients may negatively affect outcomes in the non-uterine group. If our observations are confirmed in other series, they would suggest that studies performed in STS patients should not only be stratified for LMS but also for uterine and non-uterine primary disease localisation and for gender.

## Figures and Tables

**Table 1:** Patient characteristics, *n* = 65.

	Non-Uterine LMS	%	Uterine LMS	%
	(*n* = 32)		(*n* = 33)	
Gender				
Male	13	41		
Female	19	59	33	100
Median age at diagnosis	55.5	yrs	54.4	yrs
Range	30–74		33–74	
Histology				
Spindle cell	6	19	3	9
Epitheloid	0		2	6
Pleiomorph	7	22	6	18
Unknown	19	59	22	67
Primary disease localisation				
Extremity	17	53	—	
Non-extremity	15	47	—	
Locally advanced disease				
Without distant metastases	5	16	4	12
With distant metastases	6	19	12	36
Distant Metastases (M1)				
One site, no liver	10	37	15	52
One site, liver	2	7	0	0
≥2 sites, no liver	8	30	9	31
≥2 sites, including liver	7	26	5	17
WHO PS before start chemotherapy				
0	10	31	9	27
1	21	66	23	70
2	1	3	1	3

**Table 2:** Treatment characteristics.

	Non-Uterine	%	Uterine	%
	(*n* = 32)		(*n* = 33)	
Chemotherapy				
Yes	32		33	
Induction chemotherapy (for locally advanced disease)	5		4	
No	27		29	
Chemotherapeutic regimen				
*Monotherapy *				
Doxorubicine 75 mg/m^2^, q 3 wk	17	53	20	61
Ifosfamide 9 gr/m^2^, q 3 wk	8	25	7	21
Ifosfamide 5 gr/m^2^ in 24 hrs, q 3 wk	1		0	
Epirubicine 150 mg/m^2^, q 3 wk	1		1	
Docetaxel 100 mg/m^2^, q 3 wk	1		1	
*Combination *				
Doxorubicine 75 mg/m^2 ^ + ifosfamide 9 gr/m^2^, q 3 wk	0		1	
Doxorubicine 75 mg/m^2^ + ifosfamide 10 gr/m^2^ (4 × 2.5), q 3 wk	2		1	
Doxorubicine 75 mg/m^2^ + ifosfamide 5 gr/m^2^, q 3 wk	0		1	
Doxorubicine 50 mg/m^2^ + ifosfamide 5 gr/m^2^ dg 1, q 3 wk	1		1	
Doxorubicine 60 mg/m^2^ + ifosfamide 5 gr/m^2^, q 3 wk	1		0	
				
Median number of cycles	4	(1–7)	4	(1–7)

**Table 3:** Response rate of first line chemotherapy.

Best response	Non-Uterine	%	Uterine	%
(WHO/RECIST)	(*n* = 32)		(*n* = 33)	
CR	0		1	3
PR	7	22	4	12
SD	15	47	14	42
PD	9	28	14	42
Non-evaluable	1	3	0	0

CR: complete response, PR: partial response, SD: stable disease, PD: progressive disease.
